# Potentially traumatic events, posttraumatic stress symptoms, and skin-related quality of life among adults with self-reported skin disease symptoms

**DOI:** 10.1007/s00403-024-03451-w

**Published:** 2024-11-15

**Authors:** Taylor A. Strange, Heather L. Clark, Laura J. Dixon

**Affiliations:** Department of Psychology, University of Mississippi, P.O. Box 1848, Mississippi, 38655-1848 USA

**Keywords:** PTSD, Trauma, Health psychology, Dermatology, Skin disease

## Abstract

The connection between stress and skin disease has been extensively documented; however, there are no empirical studies investigating the incidence of traumatic event exposure and posttraumatic stress (PTS) symptoms among dermatology patients. To address this gap in the literature and begin to understand the associations between PTS symptoms and skin disease symptoms, this study used a sample of adults with self-reported skin disease symptoms to examine: (1) rates of potentially traumatic event (PTE) exposure and PTS symptoms; and (2) the association between PTS symptoms and skin-related quality of life, controlling for relevant covariates. Data were collected online through Cloud Research, and participants completed a battery of self-report measures. The sample included 310 participants (68.4% female) who endorsed current skin disease symptoms. Results indicated that 47.1% of participants endorsed clinical levels of PTS symptoms. Consistent with hypotheses, greater levels of PTS symptoms were associated with worse skin-related quality of life, and this association was particularly robust for arousal-related symptoms. Results shed light on the occurrence of trauma-related experiences among individuals with self-reported skin disease and indicate a link between PTS symptoms and the perceived burden of skin disease symptoms on daily living. However, this study was cross-sectional and relied on self-report measures; therefore, findings should be interpreted with caution, particularly since diagnoses could not be verified. Replication of this work in dermatology patients is needed to further understand these connections.

## Introduction

Psychological stress is common in dermatology patients, with studies showing that 35.6% [23] to 59.8% [5] of dermatology patients report higher levels of stress compared to control samples. Numerous connections among the neuroendocrine, immune, and skin systems have been posited to explain the connection between stress and skin diseases. Albeit simplified, the impact of skin symptoms can lead to distress, and at the same time, inflammatory responses associated with stress can exacerbate skin symptomatology, thereby perpetuating a cycle [87]. In dermatology samples, higher stress has been associated with an array of adverse outcomes, including worse skin symptoms [16, 36], higher anxiety and depression [27, 58], and diminished quality of life [26, 60] across skin conditions.

Recent research has begun to clarify the relationships between specific types of stress and skin disease severity. Potentially traumatic experiences (PTEs) are adverse experiences, such as actual or threatened death, injury, or sexual violence [3]. National studies of U.S. adults have found that exposure to PTEs is common, with approximately 89.7% of individuals experiencing these events at some point in their life [44]. Although many individuals experience PTEs, only 3.9–5.6% develop posttraumatic stress disorder (PTSD) [45], which is characterized by persistent and impairing symptoms that entail re-experiencing the traumatic event (e.g., nightmares), avoidance of trauma-related stimuli (e.g., thoughts), increased negative thoughts or feelings (e.g., exaggerated self-blame), and increased arousal and reactivity (e.g., hypervigilance).

Posttraumatic stress (PTS) symptom severity has been positively correlated with chronic stress [62] and abnormally prolonged and elevated stress hormone levels [62, 70]. When exposed to PTE cues, individuals with PTSD have been found to exhibit significantly higher cortisol levels than those without PTSD who experienced a PTE [29]. Moreover, higher levels of psychological stress have been associated with higher cortisol levels, suppressed immune function, greater inflammation, and higher susceptibility to infection [19], which may negatively affect skin diseases by impairing wound healing and exacerbating existing skin conditions [87]. For instance, one study found that greater stress exposure predicted worse psoriasis severity and was associated with a dysregulated HPA axis and pro-inflammatory state among patients with psoriasis [64]. Thus, dermatology patients who have experienced PTEs or who have been diagnosed with PTSD may be particularly susceptible to deleterious physical and mental health outcomes. In fact, research has shown that PTS is associated with worse health management [86] and greater health care utilization [14]. Given that individuals with PTSD often present to medical settings [33, 57], it is important to consider how individuals with skin disease may be affected by traumatic stress.

Although many studies have demonstrated that skin disease worsens with greater stress [6, 16], little is known about the prevalence and impact of PTEs and PTSD symptoms among adults with skin disease. Due to the psychological [59], physical [56, 71], and financial costs [30] associated with PTSD, further research is needed to understand how trauma-related experiences affect individuals with skin disease. Consequently, the following aims and hypotheses were investigated in a sample of adults with skin disease symptoms. First, this study characterized the impact of self-reported skin disease symptoms on quality of life, occurrence of PTEs, and PTS symptoms. In addition, this study examined associations between PTS symptoms and the burden of self-reported skin disease symptoms on daily living (i.e., skin-related quality of life). It was predicted that PTS symptoms would be significantly associated with skin-related quality of life, after accounting for sex [9], age [34], race/ethnicity [55], marital status [65], and perceived skin symptom severity [26]. In particular, arousal and reactivity symptoms were expected to evidence a robust association with skin-related quality of life given the proposed association between physiological reactions and trauma symptoms in dermatology patients [37].

## Method

### Participants and procedure

The current study was part of a parent project that assessed mental health concerns among individuals who screened positive for self-reported skin disease symptoms. Participants were recruited through Cloud Research from Amazon’s online Mechanical Turk (MTurk) service [49]. MTurk is a crowdsourcing online platform wherein individuals are compensated to complete tasks, such as surveys [13, 49]. MTurk has been evaluated to be a reliable and efficient source for diverse clinical and non-clinical samples [52, 69] and has been used in prior research to investigate mental health concerns among individuals with dermatological symptoms [12, 26, 61]. To be eligible for this study, individuals had to: (a) be located in the United States (U.S.); (b) have completed at least 100 hits with a 90% approval rating; and (c) have passed four attention checks that were distributed throughout the survey (e.g., paying attention is important, please select “wallet” from the items below). Participants were compensated $0.10 for the initial screener and up to $3.50 upon completion of full study measures. All study protocols and procedures were approved by the university’s institutional review board.

Individuals were initially asked to complete a brief screening questionnaire assessing skin-related symptoms (*N* = 1516; [23]). Individuals who endorsed skin disease symptoms were invited to take part in the full study (*n* = 489). Of these individuals, 310 participants consented to study procedures, completed all questionnaires, passed attention checks, and provided valid responses to the symptom checklists (e.g., did not endorse the presence of all or almost all dermatological disorders). Most participants in the final sample were White (68.4%) and female (59.2%). The average age of participants in the study was 33.9 years (*SD* = 11.0). See Table 1 for a summary of sociodemographic characteristics.

**Table 1:** Demographic characteristics

		*n* (%)
Age^a^		33.9 (11.0)
Sex	Female	184 (59.4)
	Male	126 (40.6)
Race/Ethnicity^b^	White	212 (68.4)
	Black or African American	46 (14.8)
	Asian	37 (11.9)
	Hispanic, Latinx, or Spanish	19 (6.1)
	American Indian or Alaska Native	15 (4.8)
	Middle Eastern or North African	3 (1.0)
	Native Hawaiian or Other Pacific Islander	3 (1.0)
	Other	2 (0.6)
	Prefer Not to Answer	1 (0.3)
Marital status	Married/Civil Union	139 (44.8)
	Non-married relationship	110 (35.4)
	Single	61 (19.7)
Education	High school diploma or equivalent	19 (6.1)
	Vocational training	4 (1.3)
	Some college	69 (22.2)
	Associate’s degree	23 (7.4)
	Bachelor’s degree	136 (43.7)
	Some post-undergraduate education	8 (2.6)
	Master’s degree	43 (13.9)
	Doctorate degree	8 (2.6)
Employment status	Employed full-time	167 (53.9)
	Employed part-time	87 (28.1)
	Unemployed	43 (13.9)
	Retired or “Other”	13 (4.2)

*N* = 310

^a^ Mean (Standard Deviation) reported

^b^ Participants could endorse multiple items and percentages do not add up to 100

^c^ Other skin diseases identified by a small portion of the sample, such as pruritus, ulcers, urticaria, scabies, fungal skin diseases, impetigo, skin abscesses, bacterial skin diseases, cellulitis, warts, skin lesions, non-melanoma skin cancer

### Measures

#### Demographic and skin disease characteristics

Participants provided demographic information including age, sex, race/ethnicity, marital status, education level, and employment status. Skin disease characteristics were provided by a self-report questionnaire. Perceived skin disease severity was assessed with a single self-reported item that used a 0 (*normal or no symptoms*) to 10 (*very severe symptoms*) Likert-type scale. This item has been used in prior work to examine skin symptom severity among dermatology patients [8, 35, 51, 68] and adults with skin disease symptoms [26].

#### Skin-related quality of life and impairment

The Skindex-16 [18] was used to evaluate the effects of skin disease on impaired functioning and quality of life. The Skindex-16 includes 16 items that assess three domains – physical symptoms (e.g., skin condition itching), psychological/emotional symptoms (e.g., embarrassment related to skin condition), and functioning (e.g., interference in daily activities related to skin condition). In this study, individuals respond to 16 items on a six-point bipolar scale from ‘never bothered’ to ‘always bothered’, rather than a seven-point scale in the original scale due to an error in data collection; however, consistent with the established scoring procedures, each item was transformed to a 0-100 scale, with a mean score generated for each scale. Lower quality of life is indicated by higher Skindex-16 scores. The Skindex-16 was developed from the 29-item version of the Skindex [17] and has high internal consistency and demonstrates construct and content validity among dermatology patients [18]. In this study, the Skindex-16 had excellent internal consistency (α = 0.95).

#### Potentially traumatic events

Experience of potentially traumatic events (PTEs) was assessed with the Life Events Checklist for DSM-5 (LEC-5) [32, 84]. The LEC-5 contains 17 items that list potentially traumatic events outlined by DSM-5 criteria (e.g., natural disaster). The measure is scored using a 6-point nominal scale: “Happened to me,” “Witnessed it,” “Learned about it,” “Part of my job,” “Not sure,” and “Doesn’t apply.” This measure does not produce a composite or total score. To simplify the characterization of exposure, the options “witnessed it” and “learned about it” were dichotomized to form one response and are hereby referred to as “indirectly experienced.” In addition, the response options “not sure” and “doesn’t apply” were collapsed. Thus, the response options characterized in this study included: “happened to me,” “indirectly experienced,” “part of my job,” and “not sure/doesn’t apply”.

#### Posttraumatic stress symptoms

PTS symptom severity was measured with the PTSD Checklist for DSM-5 (PCL-5) [84]. The PCL-5 is a 20-item questionnaire designed to measure severity of distress and frequency of posttraumatic stress symptom severity, such as repeated, disturbing dreams of the stressful experience and difficulty concentrating, in the past month in response to a PTE [7, 84]. Participants indicate the extent to which they agree with each statement (e.g., being bothered by “repeated, disturbing dreams of the traumatic event,”) on a 5-point Likert-type scale, ranging from 0 (*Not at all*) to 4 (*Extremely*), with higher scores corresponding to more severe PTS symptomatology. The PCL-5 has four subscales that correspond to each of the PTS symptom clusters, which includes symptoms of intrusion (i.e., Cluster B, reliving memories of the event), avoidance (i.e., Cluster C, avoiding physical and introspective reminders of the traumatic event), negative alterations in cognitions and mood (i.e., Cluster D, difficulty remembering the traumatic event or increased negative emotions towards self or others), and alterations in arousal and reactivity (i.e., Cluster E, aggression, hypervigilance, sleep disturbance, etc.). All items are summed to yield a total score ranging from 0 to 80, with higher scores indicating worse PTS symptoms. Subscale scores are derived by summing the corresponding items. A cutoff score of 33 is recommended to predict a provisional DSM-5 PTSD diagnosis [11]; however, in the current study, this cutoff was used to identify individuals with clinical levels of PTS symptoms given the absence of a verified Criterion A event. That is, although participants completed the LEC-5, they responded to PCL-5 items considering a “very stressful experience” and “keeping [the] worst event in mind,” but were not asked to identify this event. In this study, excellent internal consistency was observed for the PCL-5 total (α = 0.97) and subscale scores (αs = 0.90 − 0.94).

### Data analysis

All analyses were conducted in SPSS Version 28. First, descriptive statistics and bivariate correlations were conducted. Next, three-step hierarchical regression analyses were conducted to test the hypothesis that PTS symptoms, and in particular, the arousal and reactivity cluster, would account for unique variance in skin-related quality of life, above and beyond relevant covariates. Demographic characteristics were entered in the first step and relevant variables were dichotomized given the few number of individuals in subgroups. Demographic variables included sex (0 = female, 1 = male), age, race/ethnicity (0 = White, 1 = Person of Color), and marital status (0 = single, 1 = romantic relationship). Perceived skin symptom severity was entered in the second step, and PTS symptom clusters were entered in the third step. Assumptions of linear regression were examined, and multicollinearity was observed (variance intolerance factors [VIFs] = > 5, tolerance values < 0.20) when the four PTS symptom clusters were simultaneously; therefore, separate models were conducted for PTS total and each PTS symptom cluster, wherein the relevant PTS symptom predictor was entered in Step 3. Effect sizes for hierarchical regressions were calculated (*f*^*2*^ small ≥ 0.02; medium ≥ 0.15; large ≥ 0.35; [20]). All tests were two-tailed and alpha was set at 0.05.

## Results

### Descriptive statistics and bivariate correlations

The most common skin disease currently experienced by participants was atopic dermatitis (40.3%), acne (27.1%), and rosacea (17.1%). See Table 2 for self-reported skin disease characteristics. With regard to PTEs, the majority of participants (95.5%) reported directly (77.4%) or indirectly (86.1%) experiencing at least one PTE (see Table 3). The most commonly endorsed PTE experienced directly was a transportation accident (39.4%), followed by an unwanted sexual experience (30.6%), physical assault (29.4%), and a natural disaster (26.8%). According to the PCL-5, 47.1% of the participants endorsed clinically significant levels of PTS symptoms [11, 84].

**Table 2:** Self-reported skin disease characteristics

		*n* (%)
Current skin disease(s)^a^	Eczema (atopic dermatitis)	125 (40.3)
	Acne	84 (27.1)
	Rosacea	53 (17.1)
	Facial dermatitis	48 (15.5)
	Psoriasis	45 (14.5)
	Hyperhidrosis	44 (14.2)
	Other dermatitis	41 (13.2)
	Seborrheic dermatitis	25 (8.1)
	Alopecia	19 (6.1)
	Vitiligo	16 (5.2)
	Other skin condition^b^	149 (48.0)
Pattern of skin symptoms	Chronic	109 (35.2)
	Seasonal (i.e., regularly but seasonal patterns)	82 (26.5)
	Episodic	65 (21.0)
	Acute (onsets suddenly)	46 (14.8)
	Other	8 (2.6)
Identification of skin disease	Physician or medical professional	205 (66.1)
	Self-diagnosis	99 (31.9)
	Other^c^	4 (1.3)
	Missing	2 (0.6)
Past year medical appointment for skin-related problem	Yes	241 (78.1)
	No	68 (21.9)
Type of provider	Dermatologist	178
	General physician	120
	Cosmetologist	3
	Other	6
	None	3

*Note* Current skin disease = experienced in the past four weeks; Pattern of skin symptoms = “How would you describe the pattern of your skin symptoms since you were first diagnosed? Identification of skin disease = Past year medical appointment = “In the last 12 months, have you seen a medical provider (physician, nurse, dermatologist) for a skin-related problem?”; Type of provider = “What type of provider do you see for your dermatology/skin disease?”

^a^ Participants could endorse multiple items and percentages do not add up to 100

^b^ Other skin diseases identified by a small portion of the sample, such as pruritus, ulcers, urticaria, scabies, fungal skin diseases, impetigo, skin abscesses, bacterial skin diseases, cellulitis, warts, skin lesions, non-melanoma skin cancer

^c^ Participants reported identification of skin disease diagnosis by family history and both self-diagnosis and doctor

**Table 3:** Descriptive statistics for potentially traumatic events (PTEs)

	Happened to me	Indirectly experienced	Part of my job	Not sure or does not apply
	*n* (%)	*n* (%)	*n* (%)	*n* (%)
Natural disaster (e.g., flood, tornado)	83 (26.8)	139 (44.8)	23 (7.4)	103 (33.2)
Fire or explosion	24 (7.7)	129 (41.6)	22 (7.1)	155 (50.0)
Transportation accident (e.g., car accident, boat accident, train wreck, plane crash)	122 (39.4)	148 (47.7)	24 (7.7)	70 (22.6)
Serious accident at work, home, or during recreational activity	37 (11.9)	107 (34.5)	24 (7.7)	162 (52.3)
Exposure to toxic substance (e.g., dangerous chemicals, radiation)	23 (7.4)	82 (26.5)	32 (10.3)	195 (62.9)
Physical assault (e.g., being attacked, hit, slapped, kicked, beaten up)	91 (29.4)	127 (41.0)	24 (7.7)	114 (36.8)
Assault with a weapon (e.g., being shot, stabbed, threatened with a knife, gun, bomb)	27 (8.7)	110 (35.5)	17 (5.5)	175 (56.5)
Sexual assault (e.g., rape, attempted rape, made to perform any type of sexual act through force or threat of harm)	68 (21.9)	109 (35.2)	18 (5.8)	142 (45.8)
Other unwanted or uncomfortable sexual experience	95 (30.6)	95 (30.6)	19 (6.1)	138 (44.5)
Combat or exposure to a war-zone (in the military or as a civilian)	15 (4.8)	74 (23.9)	29 (9.4)	207 (66.8)
Captivity (e.g., being kidnapped, abducted, held hostage, prisoner of war)	17 (5.5)	59 (19.0)	19 (6.1)	221 (71.3)
Life-threatening illness or injury	45 (14.5)	145 (46.8)	20 (6.5)	124 (40.0)
Severe human suffering	31 (10.0)	114 (36.8)	22 (7.1)	158 (51.0)
Sudden violent death (e.g., homicide, suicide)	16 (5.2)	121 (39.0)	27 (8.7)	158 (51.0)
Sudden accidental death	13 (4.2)	124 (40.0)	21 (6.8)	163 (52.6)
Serious injury, harm, or death you caused to someone else	19 (6.1)	66 (21.3)	16 (5.2)	219 (70.6)
Any other very stressful event or experience	107 (34.5)	110 (35.5)	22 (7.1)	119 (38.4)
% of individuals with PTE	77.4 (240)	86.1 (267)	29.0 (90)	89.4 (277)^a^
Total # of PTEs *M* (*SD*)	2.7 (2.6)	6.0 (4.7)	1.2 (2.5)	-
Total # of PTEs Median	2.0	5.0	0	-
Total # of PTEs Mode	0	0	0	-
Range of PTEs	16	17	13	-

*Note* PTEs were assessed using the Life Events Checklist for DSM-5 [32, 84]. Participants could endorse multiple items and percentages do not add up to 100.

^a^ % of participants reporting “not sure” or “does not apply” to one or more event

Means, standard deviations, ranges, and bivariate correlational analyses for perceived skin disease symptom severity, skin-related quality of life (Skindex-16), and PTS symptoms (PCL-5) are reported in Table 4. Each PTS symptom cluster was moderately associated with skin-related quality of life.

**Table 4:** Descriptive statistics and Pearson Correlations for primary study variables

	1	2	3	4	5	6	7
1. Skin Symptoms	—						
2. QOL Total	0.595	—					
3. PTS Total	0.428	0.555	—				
4. PTS Intrusion	0.413	0.514	0.951	—			
5. PTS Avoidance	0.382	0.501	0.868	0.848	—		
6. PTS Cognitions	0.394	0.516	0.971	0.887	0.809	—	
7. PTS Arousal	0.421	0.558	0.946	0.846	0.749	0.898	—
Mean	5.7	61.2	30.5	7.4	3.2	10.8	9.2
*SD*	2.3	20.3	21.9	6.1	2.5	8.0	6.5
Observed Range	0–10	17.7–100	0–80	0–20	0–8	0–28	0–24
Possible Range	0–10	16.7–100	0–80	0–20	0–8	0–28	0–24

*Note* All correlations are significant at the *p* < .001 level (two-tailed); Skin symptoms = perceived skin disease symptom severity reported on a scale of *0 no symptoms – 10 very severe symptoms*; QOL Total = skin-related quality of life (Skindex-16); PTS = Posttraumatic Stress; PCL = PTSD Checklist for DSM-5 (PCL-5); PTSD Total = PCL-5 Total Score; PTS Intrusion = PCL-5 Intrusion scale; PTS Avoidance = PCL-5 Persistent Avoidance; PTS Cognitions = PCL-5 Negative Alterations in Cognition and Mood; PTS Arousal = PCL-5 Alterations in Arousal and Reactivity; *SD* = standard deviation

### Hierarchical regression analyses

See Table 5 for results of the hierarchical regression analyses. In Step 1, demographic characteristic accounted for 8.4% of the variance (*f*^*2*^ = 0.09, small effect). In Step 2, perceived skin disease severity accounted for an additional 29.4% of the unique variance in the model (*f*^*2*^ = 0.30, medium-large effect). Finally, in Step 3, the addition of each PTS symptom predictor accounted for unique variance in skin-related quality of life – above and beyond variance attributed to relevant demographic factors and perceived skin disease severity. Specifically, PTS total symptoms added 10.0% unique variance and the overall model including PTS total symptoms accounted for 47.8% of the total variance in skin-related quality of life, indicating a large effect (*f*^*2*^ = 0.92). Each of the PTS symptom clusters added between 7.9 and 10.1% unique variance and accounted for 45.6–47.9% of the total variance in skin-related quality of life. Thus, the addition of each PTS symptom predictor demonstrated medium effects (*f*^*2*^ = 0.15 − 0.20), while a large effect was observed for each of the overall models (*f*^*2*^ = 0.84–92). Consistent with hypothesis, the strongest effect was observed for the association between arousal symptoms and skin-related quality of life (Δ*R*^*2*^ = 0.101, *f*^*2*^ = 0.20).

**Table 5:** Hierarchical regression analyses examining PTS symptoms in skin-related quality of life

	ΔR^2^	F	B		SE		*p*
Step 1	0.08	*F* (4, 304) = 6.93					< 0.001
Sex			6.59		2.26		0.004
Age			0.22		0.10		0.037
Race/Ethnicity			6.03		2.38		0.012
Marital Status			9.37		2.83		0.001
Step 2	0.29	*F* (5, 303) = 36.75					< 0.001
Skin Symptom Severity			4.92		0.41		< 0.001
Step 3							
PTS Total	0.10	*F* (6, 302) = 46.02	0.33		0.04		< 0.001
PTS Intrusion	0.08	*F* (6, 302) = 42.26	1.04		0.16		< 0.001
PTS Avoidance	0.08	*F* (6, 302) = 42.77	2.50		0.37		< 0.001
PTS Cognitions	0.09	*F* (6, 302) = 43.54	0.82		0.12		< 0.001
PTS Arousal	0.10	*F* (6, 302) = 46.26	1.11		0.14		< 0.001

*Note* Statistics reported correspond to the model in which the factors were entered. Step 3 examined each PTS predictor in a separate hierarchical regression model. Sex is coded 0 = female, 1 = male; Race/Ethnicity is coded 0 = White, 1 = Person of color; Marital Status is coded 0 = single; 1 = romantic relationship; Skin Symptom Severity = perceived skin symptom severity; PTS = Posttraumatic Stress Symptoms; PTS Intrusion = PCL-5 Intrusion scale; PTS Avoidance = PCL-5 Persistent Avoidance scale; PTS Cognitions = PCL-5 Negative Alterations in Cognition and Mood; PTS Arousal = PCL-5 Alterations in Arousal and Reactivity

## Discussion

Despite extensive literature demonstrating the association between stress and skin disease [1, 16, 46, 63], to the authors’ knowledge, no study had investigated the prevalence of PTEs and PTS symptoms in dermatology samples, nor evaluated the unique role of PTS symptoms in skin-related quality of life. Given the prevalence of PTEs and PTSD in the general population, identifying rates of PTS symptoms in patients with skin diseases is important for identifying relevant resources, detecting mental health symptoms in dermatology patients, and informing research priorities. Consequently, the current findings add to the understanding of specific stress-related factors that may influence the experience of skin disease by examining the occurrence of PTEs, PTS symptoms, and associations with skin-related quality of life.

The first aim was to characterize PTEs and PTS symptoms in a sample of individuals with self-reported skin disease symptoms. On average, skin-related symptoms appeared to have a moderate impact on participants’ quality of life, which is consistent with studies examining quality of life in samples of dermatology patients [15, 17, 47]. Regarding PTEs, 96.5% of the sample reported exposure to at least one PTE, which is slightly higher than rates observed in the U.S. population (i.e., 89.7%; [44]). Interestingly, the most endorsed event in this sample was motor vehicle accidents, whereas physical or sexual assault, death of a loved one, and natural disaster are the most common events reported at the national level [44]. However, PTEs have been found to vary across gender and racial/ethnic groups (e.g., [80]), and consistent with the current findings, researchers have illustrated that other injuries or shocking experiences, such as a serious car accident may be more likely to occur within an individual’s lifetime [39]. Finally, nearly half of participants endorsed elevated levels of posttraumatic stress symptoms, indicating high levels of distress in this sample. Indeed, rates of PTSD, and psychopathology more generally appear to be higher in MTurk samples. For instance, van Stolk-Cooke and colleagues [73] found 19.8% of MTurk participants endorsed clinical levels of PTSD, and higher levels of psychological symptoms have been reported among MTurk samples compared to community and college samples [4]. Thus, these higher rates of PTS symptoms may reflect greater distress more broadly, rather than specifically PTSD. Given the strong link between PTSD and inflammatory diseases [10, 75], additional research in dermatology samples is needed to further understand the base rates of PTEs and PTSD in dermatology patients.

Large effects were observed for the regression models. Although perceived skin symptom severity was a large contributor to understanding skin-related quality of life, PTS symptoms explained additional, unique variance in skin-related quality of life. In particular, the strongest association was observed for the relationship between the PTS arousal and reactivity cluster and skin-related quality of life. These results suggest that increased levels of hyperarousal and reactivity, such as sweating, may negatively influence the impact of skin disease symptoms. PTSD has increasingly been linked to heightened inflammation where the immune system is in overdrive, releasing excess inflammatory chemicals throughout the body even when the body is not in immediate danger [75]. Consequently, individuals diagnosed with PTSD may be at higher risk of developing or exacerbating, certain physical health conditions [59]. For instance, inflammatory responses have been shown to be exacerbated by trauma-related symptoms and increased rates of comorbidity between PTSD and chronic health conditions, such as chronic hives [40], rheumatoid arthritis [50], and asthma [82] have been observed. These findings contribute to research suggesting greater severity of posttraumatic stress symptoms occurs alongside worsened physical health concerns [6, 19]. Cumulatively, these findings suggest that greater perceived severity of health conditions, including skin diseases, may be affected by the severity of PTS symptoms. Additional studies are needed to further evaluate the impact of PTS on skin disease burden and examine biological (e.g., inflammatory markers) and psychological processes (e.g., anxiety sensitivity, pain catastrophizing) that may contribute to the association between PTS and skin symptoms across different types of dermatological disorders.

Although this study provides a novel contribution to the literature, several limitations should be considered when interpreting these findings. First, participants were recruited online, and diagnoses were not verified. Future work should consider the use of a structured diagnostic interview to verify the Criterion A event and diagnoses of PTSD and medical records to confirm skin disease diagnoses. Prior research has demonstrated good agreement between self-reported medical diagnoses and clinically determined diagnoses for chronic health conditions, such as hypertension [2], diabetes [54], heart disease [54], cancer [53, 54], and individuals with severe asthma [78]. However, a previous study showed mixed agreement for dermatology diagnoses and showed patterns of misdiagnosis and underestimates of the prevalence of certain dermatological disorders (e.g., psoriasis, acne) [42]. Second, participants in this study endorsed a range of skin disease diagnoses. In comparing skin disease symptoms reported by this sample to national findings, the most prevalent skin diseases were eczema, acne and rosacea, whereas noncancerous skin growths, cutaneous infections, and viral and fungal diseases were found to be the most common skin diseases reported to insurance companies in the U.S. in 2013 [48]. Thus, the findings may not fully generalize to patients seen through the U.S. health care system, particularly as the disorders commonly observed in this study are often identified as psycho-dermatological disorders, which are associated with higher rates of psychological symptoms [25, 41]. Third, this study is cross-sectional and causality cannot be inferred. Additional research is needed to examine the directionality of these associations and to identify psychological and biological risk factors for the development of PTSD. Research indicates a bidirectional relationship between PTSD and heightened inflammation, suggesting that inflammation may increase susceptibility to PTSD after trauma, and that PTSD may lead to altered inflammatory responses [75]. Similar to the current findings, researchers have observed that higher levels of PTSD have been associated with other diseases rooted in inflammatory responses, such as more severe asthma [82], worsened symptoms of rheumatoid arthritis, GI issues, and respiratory illness [43]. These findings suggest that similar symptom patterns may be observed for those with dermatological disorders, indicating that longitudinal study designs are needed to investigate the trajectory of skin disease symptoms following a PTE. Last, this sample comprised predominately White females, and results may not generalize to all patients presenting with skin disease concerns. Given that patients presenting to dermatology clinics are primarily White and female [79], these findings may inform the presentation of patients in outpatient dermatology settings.

In conclusion, PTEs and clinically significant symptoms of PTS were found to be common among individuals with self-reported skin disease and such symptoms were associated with worse skin-related quality of life. Clinically, these results indicate the potential benefit of screening dermatology patients for PTSD symptoms. Although psychological concerns such as depression and anxiety are highly prevalent among dermatology patients [83], research suggests that resident training in mental health screening [74], dermatologist-performed mental health screens [76, 77], and detection of these possible conditions are low [67]. Calls for increased attention to the importance of mental health screening in dermatology settings have existed for many years [21], though even recent guidelines for clinicians fail to address the potential impact of posttraumatic stress symptoms specifically on dermatology presentation [24, 28]. In other outpatient settings, identifying trauma-specific symptoms or PTSD is also low for non-VA service seeking patients [22, 33, 72]. Detecting PTSD symptoms is critical for connecting patients with relevant information and referrals. Importantly, there are several evidence-based psychological interventions for PTSD, such as Cognitive Processing Therapy [66] and Prolonged Exposure Therapy [31], which emphasize changing maldaptive cognitive and behavioral patterns related to the traumatic event(s). Such interventions have been found to reduce risk for type II diabetes and hypertension [81], and consistent with prior studies examining cognitive behavioral treatments in dermatology samples, they may also alleviate dermatology symptoms [38, 85].

## Data Availability

No datasets were generated or analysed during the current study.
